# Electromechanical therapy in diabetic foot ulcers patients: A systematic review and meta-analysis

**DOI:** 10.1007/s40200-023-01240-2

**Published:** 2023-06-08

**Authors:** Ayeshmanthe Rathnayake, Apoorva Saboo, Venkat Vangaveti, Usman Malabu

**Affiliations:** 1 Translational Research in Endocrinology and Diabetes, College of Medicine and Dentistry, James Cook University, Townsville, 4811 Australia; 2 Department of Diabetes and Endocrinology, Townsville University Hospital, Douglas, Australia

**Keywords:** Diabetes, Diabetic foot ulcer, Electromechanical therapy, Adjunctive therapy, Systematic review, Meta-analysis

## Abstract

**Purpose:**

Diabetic foot ulcer (DFU) is one of the most devastating and troublesome consequences of diabetes. The current therapies are not always effective because of the complicated aetiology and interactions of local and systemic components in DFU. However, adjunctive therapy (electromechanical therapy) has become the latest modality in recent years, although there is a lack of significant research to support its utilization as a treatment standard. The purpose of this systematic research was to review the literature on the application of electromechanical therapies in the healing of DFUs.

**Methods:**

For this systematic review, we searched PubMed, Medline, EmBase, the Cochrane library, and Google Scholar for the most current research (1990–2022) on electromechanical therapies for DFUs. We used the PICO method (where P is population, I is intervention, C is comparator/control, and O is outcome for our study) to establish research question with the terms [Electromechanical therapy OR Laser therapy OR photo therapy OR Ultrasound therapy OR Shockwave therapy] AND [diabetic foot ulcers OR diabetes] were used as search criteria. Searches were restricted to English language articles only. Whereas, Cochrane handbook of “Systematic Reviews of Interventions” with critical appraisal for medical and health sciences checklist for systematic review was used for risk of bias assessment. There were 39 publications in this study that were deemed to be acceptable. All the suitably selected studies include 1779 patients.

**Results:**

The meta-analysis of 15 included research articles showed the overall effect was significant (P = 0.0002) thus supporting experimental groups have improvement in the DFUs healing in comparison to the control group.

**Conclusion:**

This systematic review and meta-analysis revealed electromechanical treatments are significantly viable options for patients with DFUs. Electromechanical therapy can considerably reduce treatment ineffectiveness, accelerate healing, and minimize the time it takes for complete ulcer healing.

**Supplementary Information:**

The online version contains supplementary material available at 10.1007/s40200-023-01240-2.

## Introduction

Diabetes is an emerging epidemic with rising incidence, morbidity, and death [1]. Diabetic Foot Ulcer (DFU) is one of the most devastating and troublesome consequences of diabetes and the most significant predictor for lower-extremity amputations [2]. DFU is frequently linked to infection, peripheral neuropathy, and peripheral vascular disease. Nearly 80% of nontraumatic amputations are caused by DFUs, which account for around 35% of the patients in diabetes clinics [2]. Approximately 6% of people worldwide have DFU, and the disease can have up to a 77% five-year mortality rate [2, 3]. According to the International Diabetes Federation, the number of persons with diabetes has constantly increased; currently, there are 643 million cases worldwide, and by 2045, there will be 783 million cases [4].

It is crucial to research techniques and therapies to lessen the burden of the disease in a productive and economical manner since managing DFU continues to be a significant therapeutic problem on a global scale. Due to poor leukocyte chemotaxis and phagocytosis, diminished macrophage activity in the wound matrix, decreased collagen synthesis and deposition, and reduced growth factor release, wound recovery in diabetes patients is often slower than in healthy persons [5, 6]. Diabetes patients have a poor capacity to heal wounds, which makes managing the illness more challenging. Therefore, the development of a treatment for DFUs should take into account a multidisciplinary approach that includes glycaemic management, daily local care, antimicrobials, antiseptics, surgical revascularization, and engineered biological tissues [6, 7].

Surgery debridement, dressings, pressure offloading, vascular assessment, infection treatment, glycaemic control, and patient education make up the standard of care for DFUs [8, 9]. These treatments are not always successful because of the complex aetiology and interaction of local and systemic factors. As a result, it requires a variety of time and cost periods to support the healing process [10]. An optimal adjuvant therapy has yet to be established, which is urgently needed for DFU healing [11]. According to a number of earlier research, nonpharmacological treatments such electrical stimulation [12], low-level laser therapy (LLLT), hyperbaric oxygen therapy [13], and foot off-loading may also be helpful in the healing of DFUs. Additionally, it has been proposed that the use of ultrasound, light therapy, and electrical stimulation will hasten the healing of DFUs by promoting the migration of different cell types and improving wound perfusion [14, 15]. However, an ideal adjuvant therapy has not yet been identified, which is urgently required for the wound healing of DFUs [11]. Due to the dearth of high-quality data that provides solid evidence to support their clinical use, there is a clear need for evidence to substantiate the use of electromechanical therapies (laser therapy, phototherapy, ultrasound therapy, and shockwave therapy) in the management of DFUs [16].

In the current study, we set out to comprehensively review the literature on the application of electromechanical therapies in the healing of DFUs, synthesise the results using meta-analysis of randomised controlled trials (RCTs), and provide clinical guidelines and evidence-based recommendations for the treatment of DFUs in the future.

## Material and methods

For this systematic review in November 2022, we searched PubMed, Medline, EmBase, the Cochrane library, and Google Scholar for the most current research (1990–2022) on electromechanical treatments for DFUs. PICO format was followed to design the research question as it is the recommended method by Cochrane and PRISMA guidelines. The terms [Electromechanical therapy OR Laser therapy OR photo therapy OR Ultrasound therapy OR Shockwave therapy] AND [diabetic foot ulcers OR diabetes] were used as search criteria. Searches were restricted to English language articles only. The relevancy of the titles, abstracts, and keywords was checked by two independent reviewers. Publications having titles or abstracts that complied with the requirements for this systematic review were chosen for a more thorough examination. To discover whether there were any other studies that were pertinent, we also went through the reference tracking of bibliographies and manual searches during the first search. The titles and abstracts were evaluated for inclusion by the writers independently. Only studies that satisfied the inclusion criteria were deemed eligible after being located utilising the PRISMA technique and critical appraisal tools (https://jbi.global/critical-appraisal-tools) (Table 1). The risk of bias of included studies was assessed by using an assessment tool of the “Cochrane Handbook for Systematic Reviews of Interventions version” with critical appraisal for medical and health sciences checklist for systematic review.

**Table 1:** Inclusion and exclusion criteria for studies

PICO components	Inclusion criteria	Exclusion criteria
Population	Diabetic patients with foot ulcers	Patients without diabetic foot ulcers
Intervention	Studies on the electromechanical therapies in diabetic foot ulcers	Anything not including the listed topics regarding electromechanical therapies in diabetic foot ulcers
	Peer reviewed research with all types of study designs (such as quantitative, qualitative, and mixed methods)	Anything other than peer-reviewed articles and literature such as reviews, blogs, books chapters, websites content, and more
	Published from 1990 to 2022	Published before 1990
	Original article	Reviews
	Randomized control trials	Meta-analysis/systemic reviews
	English language research	Publications in languages other than English
Comparator/Control	Placebo/ control group with other electromechanical therapy	Study without any control group
Outcome	Improvement in the diabetic foot ulcers with electromechanical therapies	Studies reporting no outcome

The data of patients, their age, sex, type of therapy/intervention, duration was extracted. Depending upon the availability of data in the studies we used Standardized Mean Difference (SMD). The statistical analysis (meta-analysis) was performed using Review Manager 5.4 and a 95% confidence interval. Based on the heterogeneity between the studies we selected random or fixed-effect model for meta-analysis. To determine the entire cumulative impact, forest plots were curated.

## Quality assessment

The assessment tool covers 7 domains: random sequence generation (selection bias), allocation concealment (selection bias), blinding of participants and personnel (performance bias), blinding of outcome assessment (detection bias), incomplete outcome data (attrition bias), selective reporting (reporting bias), and other biases. Bias was assessed as “low risk,” “high risk,” or “unclear risk.”

## Results

**Fig. 1:**
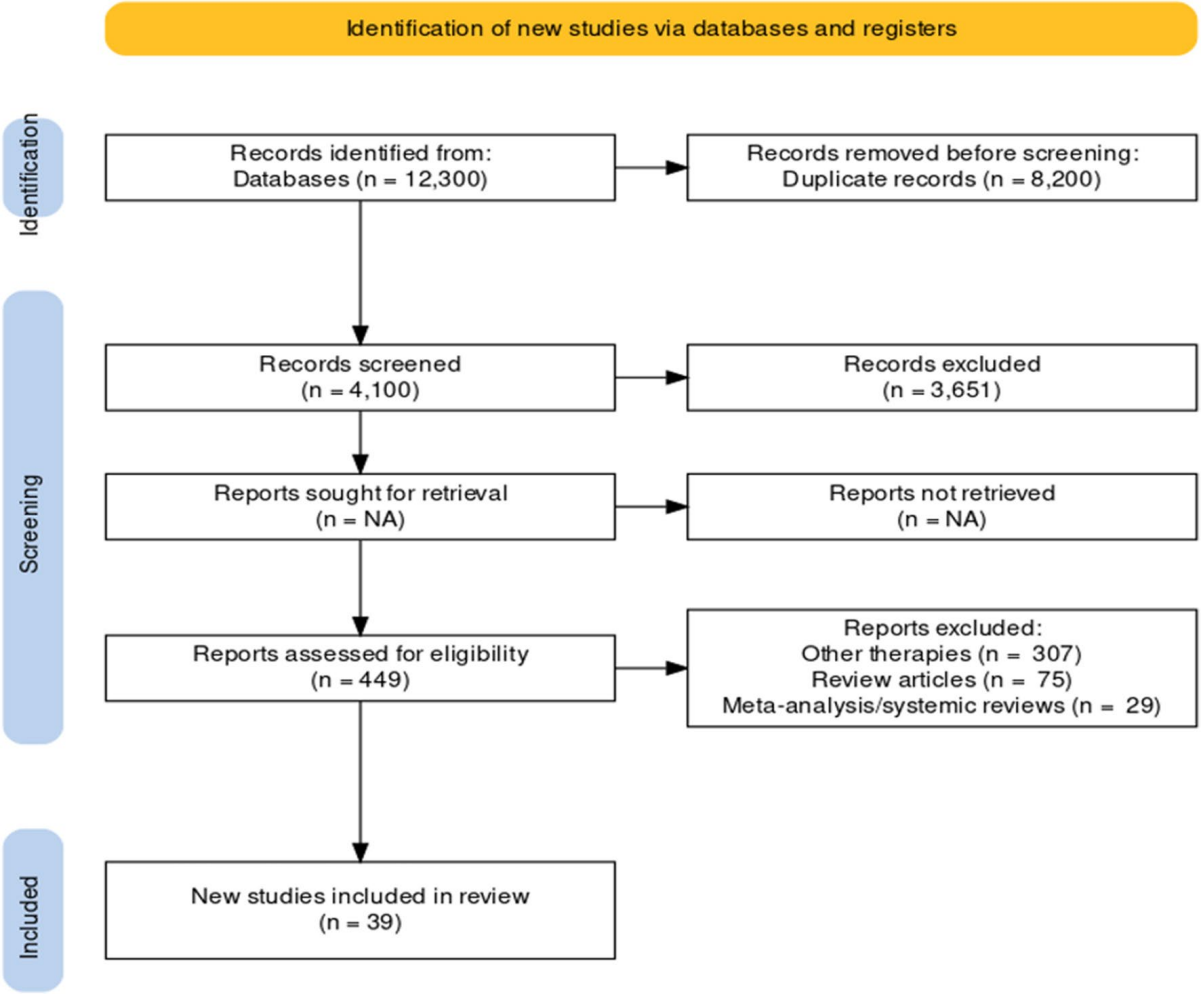
The PRISMA flow chart of the literature selection for the meta-analysis. After eliminating any obviously irrelevant information, the authors separately reviewed the research abstracts and full texts to choose which publications to include based on the inclusion and exclusion criteria (Table 1). Any issues or disagreements were discussed by all writers and were resolved

Following the PRISMA guidelines and critical appraisal tools to ensure the quality and consistency of the identified articles, several criteria were used for article eligibility as described in Table 1. After the initial search, 8200 duplicate articles from all researched databases were deleted. Further, 3651 papers were removed from the research after their titles and abstracts were examined. The remaining 449 articles were reviewed and selected by the principal author and co-author based on the set inclusion and exclusion criteria. This study comprised 39 papers that were determined to be eligible (Fig. 1).

**Fig. 2:**
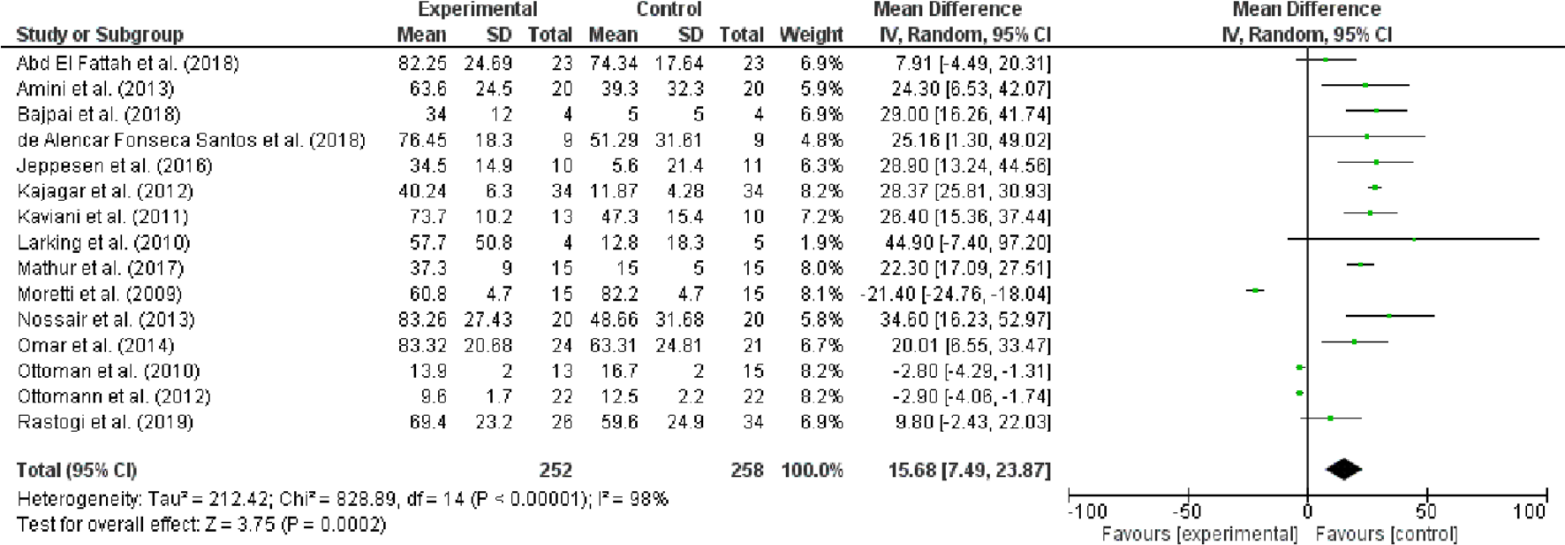
Forest plot showing improvement in the mean effects for experimental (electromechanical therapies) compared to control/placebo groups of diabetic foot ulcer patients

The selected studies include 1779 patients with DFUs. Most of the selected studies have been published in USA (n = 8), followed by Brazil (n = 5) and Egypt (n = 4) as presented in Table 2. While 11 studies reported the use of laser therapy for the treatment of DFUs, 10 studies reported shockwave therapy, 8 studies reported stimulation therapy, 7 reported ultrasound therapy and 3 reported light/phototherapy (Table 2). The mean difference (15.68) for these studies also showed significant difference among experimental and control groups (95% CI, 7.49, 23.87). The overall effect was significant (P = 0.0002) that indicates experimental groups have improvement in the DFUs healing compared to control group. Fifteen studies in the forest plot compared the electromechanical therapies vs placebo/control groups that showed significant difference (P < 0.00001) in heterogeneity among the groups with 98% I^2^ value (Fig. 2)*.*

**Table 2:** Demographic characteristics of selected studies

Sr. No	Author Year	Country	Study type	General characteristics	Patients	Type of Therapy	Duration of intervention	Findings
1	Haze, Gavish [55]	Israel	Randomized, double-blind, sham-controlled study	age = 63 ± 11 years, male:female 13:7	20	Photobiomodulation laser therapy	12 weeks	In addition to regular therapy, photobiomodulation at home may be useful for the treatment of severe DFUs in fragile patients with co-morbidities. This is especially pertinent during these times of social estrangement
2	Gao, Chen [56]	China	Randomized control trial	Age = 35 to 70 years, 57 males and 43 females	100 (50 experimental group and 50 control group)	ultrasonic debridement	4 weeks	In comparison to patients receiving Kangfuxin liquid, those with DFUs receiving the combination of ultrasonic debridement and cortex phellodendri compound fluid had better clinical efficacy, smaller ulcer areas, higher healing rates, and a lower rate of positive bacterial cultures without experiencing an increase in adverse events
3	Zulbaran-Rojas, Park [57]	USA	Double-Blinded Randomized Control Trial	Age = 18 to 85 years, 21 males and 12 females	33	Electrical Stimulation	4 Weeks	Early findings on the viability, acceptability, and efficacy of electrical stimulation as an additional treatment to hasten wound healing in patients with mild to severe peripheral artery disease and chronic DFUs are presented
4	Lázaro-Martínez, Álvaro-Afonso [58]	Spain	Randomized controlled trial	Age = 50 to 80 years, 48 males and 3 females	51 (24 in surgical debridement group and 27 in ultrasound-assisted wound (UAW) debridement group)	Ultrasound-Assisted Wound Debridement (UAW)	6 weeks	Compared to the surgical group, atherosclerotic burdens were considerably lower in the UAW debridement group. Time to recovery was also much quicker in the UAW group (9.7 ± 3.8 weeks) than with the surgical group (p = 0.04)
5	Vitoriano, Mont’Alverne [59]	Brazil	Comparative, quantitative approach study	Age = 59 ± 6.2 years, 6 males and 6 females	12	laser and LED influence	Twice a week with ten consultations	Both the neuropathic signs and symptoms and tissue healing improved with the two treatment modalities; however, the laser shown a faster rate of speed than the LED
6	Rastogi, Bhansali [60]	India	Randomized, Double-Blind, Sham-Control Study	Age = 18 to 60 years,	58	Low-Frequency, Noncontact Airborne Ultrasound Therapy	4 weeks	When used in conjunction with regular wound care, airborne low-frequency ultrasound treatment enhances and speeds up the healing of chronic neuropathic DFU
7	Abd El Fattah, Shaaban [61]	Egypt	Randomized clinical trial	Age = 35 to 67 years, 24 males and 22 females	46	ultrasound‑assisted debridement	12 weeks	The combination of normal wound care and low-frequency ultrasound (LFU) debridement promotes wound healing and reduces wound infection
8	Bajpai, Nadkarni [62]	USA	Double blind, randomized controlled study	Age = 57.6 ± 8.9 years, 4 males and 4 females	8	Non-thermal, non-cavitational ultrasound	12 weeks	Examining the value of the novel M1/M2 score can help track the progress of wound healing in DFUs because it is non-thermal and non-cavitational
9	Michailidis, Bergin [63]	Australia	Randomised controlled trial	NA	10	low frequency ultrasonic debridement versus non‑surgical sharps debridement	6 weeks	When compared to low frequency ultrasonic debridement (117.6 days ± 40.3), ulcers treated non-surgically with sharps debridement healed more quickly (61.6 days ± 24.4). Both groups' quality of life was seen to increase as the ulcers healed and the discomfort subsided as the ulcers got better
10	Snyder, Galiano [64]	USA	Controlled, double-blinded, randomised phase III clinical trials	Age = 59.9 ± 10.0 years, 269 males and 67 females	336 (172 patients treated with active therapy and 164 managed with a sham device)	Focused shockwave therapy	12 weeks	For neuropathic DFU that do not respond to conventional therapy alone, shockwave therapy is an efficient therapeutic technique when combined with standard care
11	de Alencar Fonseca Santos, Campelo [33]	Brazil	Randomized controlled trial	Age = 30 to 59 years	18	Low-Power Light Therapy	4 weeks	When compared to the Control Group, the Laser Group demonstrated a considerable improvement in the tissue healing index, with a statistically significant difference (p < 0.013). The use of low-level laser therapy (LLLT) to chronic lesions on a diabetic foot quickly advanced the tissue restoration process
12	Tantawy, Abdelbasset [65]	Egypt	Randomized controlled trial	Age = 50 to 60 years, 51 males and 14 females	65	helium–neon laser therapy and infrared laser therapy	8 weeks	T This study shows that infrared laser treatment and helium–neon laser therapy have comparable short-term effects for treating DFUs (up to 8 weeks). Laser treatment for DFUs is effective after eight weeks
13	Asadi, Torkaman [66]	Iran	Randomized controlled trial	Age = 60.8 ± 5.5 years, 14 males and 10 females	24	low-intensity cathodal direct current	4 weeks	Hypoxic inducible factor-1α (HIF-1α) and vascular endothelial growth factor (VEGF) are released in ischemic DFUs (DFUs) in a favorable manner by low-intensity cathodal direct current
14	El Rasheed, Mahmoud [67]	Egypt	Randomized controlled trial	Age = 45 to 60 years,13 males and 17 females	30 (15 in pulsed electromagnetic fields group and 15 in Infra-red laser therapy group)	Pulsed electromagnetic fields versus laser therapy	4 weeks	Two efficient and advised techniques for treating infected DFUs are pulsed electromagnetic fields and infrared laser. Regarding the same treatment settings, laser therapy is superior for wound regeneration
15	Srilestari, Nareswari [68]	Indonesia	Controlled randomized clinical trial	Age = 53 to 57 years, 17 males and 19 females	36	laser-puncture and conventional wound care	4 weeks	DFUs can be treated more quickly with a combination of laser penetration and traditional wound care
16	Mathur, Sahu [32]	India	Randomized placebo-controlled study	Age = 49 to 54 years, male:female 2:1	30 (15 in LLLT group and 15 in control group)	Low-level laser therapy	6 weeks	In the LLLT group, the percentage ulcer area decrease was 37 ± 9%, compared to 15 ± 5.4% in the control group (p < 0.001). This study demonstrated the value of LLLT as a complementary therapy for the treatment of DFUs
17	Jeppesen, Yderstraede [69]	Denmark	Prospective randomized trial	Age = 65.3 ± 12.9 years, 16 males and 7 females	23 (11 in the intervention group and 12 in the control)	Extracorporeal shockwave therapy	3 weeks	At 7 weeks, the intervention group saw a 34.5% reduction in ulcer area compared to the control group's 5.6% (p = 0.387). ESWT may help with tissue oxygenation and ulcer healing
18	Carvalho, Feitosa [35]	Brazil	Randomized controlled trial	Age 40 to 70 years	32	Low-level laser therapy	30 days	Low-level laser therapy was successful in reducing pain and accelerating the healing process of diabetic foot tissue, whether it was used alone or in conjunction with Calendula officinalis oil
19	Feitosa, Carvalho [51]	Brazil	Randomized controlled trials	NA	16 (8 in control group and 8 in low-level laser therapy with a pulsed wave form, visible ray group)	Low-Level Laser Therapy (LLLT)	30 days	T Compared to the control group, there was a statistically significant reduction in the size of the wound (p < 0.05). The treated group's discomfort was also said to have significantly improved. Regarding the tissue regeneration of ulcers in a diabetic foot, low-level laser therapy appears to be an effective, practical, painless, and inexpensive procedure
20	Sandoval Ortíz, Herrera Villabona [70]	Colombia	Randomized controlled clinical trial	Age = 59.3 ± 11.8 years, 12 males and 16 females	28	Low level laser therapy and high voltage stimulation	16 weeks	The results of this trial did not show any extra benefits of high voltage pulsed current or low-level laser therapy over standard wound care (SWC) for the healing of diabetic ulcers
21	Liani, Trabassi [71]	Italy	Randomized controlled trial	NA	25	pulsatile electrostatic field	NA	Patients with type 2 diabetes mellitus who use the technique pulsatile electrostatic field have reported changes in their metabolic processes and the rate at which ulcers heal
22	Mohajeri-Tehrani and Annabestani [72]	Iran	Randomized controlled trial	Age = 40 to 60 years, 17 males and 3 females	20	low-intensity direct current	12 days	Applying low-intensity electrical stimulation boosts the production of nitric oxide (NO) and vascular endothelial growth factor (VEGF), which may enhance tissue temperature and blood flow, promoting wound healing in DFUs
23	Omar, Alghadir [30]	Saudi Arabia	Randomized controlled trial	Age = 56.59 ± 7.35, 27 males and 11 females	38 (ESWT group and control group)	Shock wave therapy	4 weeks	With no negative effects, the median time needed for ulcer healing and the size of the wound were both significantly reduced in ulcers treated with ESWT. Therefore, the ESWT is recommended as a complementary therapy for persistent diabetic wounds
24	Nossair, Eid [73]	Egypt	Randomized controlled trial	Age = 40 to 70 years, 29 males and 11 femals	40 (shock wave group and control group)	Shock Wave Therapy	3 months	Comparing the shock wave group to the control group, a substantial reduction in wound surface area and an increase in the rate of epithelialization were seen
25	Ottomann, Stojadinovic [74]	Germany	Randomized controlled trial	Age = 53 ± 17 years, 32 males and 12 femals	44 (22 in defocused ESWT group and 22 in burn wound debridement/topical antiseptic therapy group)	Extracorporeal shockwave therapy	12 weeks	After debridement and topical antiseptic therapy, one defocused shock wave treatment was administered to the superficial second-degree burn site, and this greatly expedited epithelialization
26	Kajagar, Godhi [75]	India	Randomized controlled trial	Age = 50.94 ± 8.11 years, male:femal 2:1	68 (34 in LLLT group with conventional Therapy and 34 in control group with conventional therapy alone)	Low Level Laser Therapy	4 weeks	The percentage ulcer area decrease in the experimental group was 40.24 ± 6.30 mm^2^ while in the control group it was 11.87 ± 4.28 mm^2^ (p < 0.001, Z = 7.08). In addition to traditional therapy, low-level laser therapy is helpful in the treatment of DFUs (DFU)
27	Kaviani, Djavid [27]	Iran	Randomized clinical trial	Age = 60.2 ± 9 years, 12 males and 6 females	23 (13 LLLT group and 10 placebo group)	Low Level Laser Therapy	4 weeks	Chronic DFUs can heal more quickly with LLLT, and it is possible that LLLT could reduce the amount of time necessary for full recovery
28	Landau, Migdal [76]	Israel	Placebo-controlled double-blind study	Age = 62.9 years, 11 males and 5 females	16 (10 treatment group and 6 placebo group)	Visible Light-Induced Healing	12 weeks	Broadband (400–800 nm) (400–800 nm) Leg or foot ulcers might be effectively treated with visible light
29	Wang, Wu [77]	Taiwan	Randomized trial	Age = 20 to 88 years,	77 (39 in ESWT group and 38 in HBOT group)	Extracorporeal Shockwave Treatment and hyperbaric oxygen therapy	3 weeks	In the treatment of persistent DFUs, ESWT is superior to HBOT. In comparison to HBOT, ESWT-treated ulcers in chronic DFUs significantly improved in blood flow perfusion rate and cell activity, resulting in superior ulcer healing
30	Petrofsky, Lawson [29]	USA	Longitudinal randomized study	Age = 48.4 ± 14.6 years	20	electrical stimulation	4 weeks	For the treatment of chronic diabetic foot wounds, local dry heat and electrical stimulation (ES) are effective together; however, local heat would seem to be an important component of this therapy because ES by itself has not resulted in much healing in previous studies
31	Moretti, Notarnicola [78]	Italy	Randomized, prospective, controlled study	Age = 30 to 70 years, 16 males and 14 femals	30 (ESWT group and control group)	Shock wave therapy	20 weeks	The healing times were 60.8 and 82.2 days, respectively, and after 20 weeks of treatment, 53.33% of the ESWT-treated patients had completely closed their wounds as opposed to 33.33% of the control patients (p < 0.001)
32	Minatel, Frade [28]	Brazil	Randomized Controlled Trial	Age = 47 to 87 years,	14	Phototherapy	4 weeks	When 660 and 890 nm light are combined, diabetic ulcers that have not responded to conventional treatments can quickly granulate and heal
33	Wang, Kuo [79]	Taiwan	Randomized Controlled Trial	Age = 58.6 ± 12.6 years	70 (34 in extracorporeal shockwave treatment group and 36 in hyperbaric oxygen therapy group)	Extracorporeal Shockwave Treatment	NA	In terms of the overall findings, the extracorporeal shockwave therapy (ESWT) group indicated that 31% of cases were entirely cured, 58% had improved, and 11% had remained unaltered. HBO does not seem to be as effective as ESWT in treating persistent DFUs
34	Saggini, Figus [80]	Italy	Randomized Trial	Age = 24 to 79 years	40 (30 in extracorporeal shock waves treatment group and 10 in regular dressings treatment group)	Extracorporeal Shockwave Therapy	8 weeks	ESW therapy appears to be a secure, workable, and affordable treatment for lower extremity chronic ulcers
35	Kavros, Miller [81]	United States	Randomized controlled trial	Age = 72 to 82 years, 54 males and 16 females	70	Low-Frequency Ultrasound therapy	12 weeks	When MIST Therapy was used in conjunction with the accepted form of wound care, the rate of healing of cutaneous foot and leg ulcerations in patients with chronic critical limb ischemia significantly increased
36	Petrofsky, Lawson [82]	USA	Randomized trial	Age = 52 to 82 years	29	Local Versus Global Heat	4 weeks	Blood flow increased somewhat in the middle of the wound after electrical stimulation but nearly quadrupled on the edge and outside of the incision
37	Peters, Lavery [83]	USA	Randomized clinical trial	Age = 59.9 ± 7.0 years	40	Electrical Stimulation	12 weeks	When used in conjunction with appropriate off-loading and local wound care, electric simulation accelerates wound healing
38	Baker, Chambers [12]	USA	Randomized controlled trial	Age = 40 to 82 years, 55 males and 25 females	80	Electrical Stimulation	4 weeks	For individuals with diabetes and open ulcers, daily electrical stimulation with a brief pulsed, asymmetric biphasic waveform improved healing rate
39	Lundeberg, Eriksson [84]	Sweden	Controlled study	Age = 66 ± 7.9 years, male:female 13:18	64	Electrical Nerve Stimulation	12 weeks	According to this study, after 12 weeks of therapy, there were significant differences (p 0.05) between the electrical nerve stimulation group and the placebo group in both the ulcer area and the cured ulcers

**Fig. 3:**
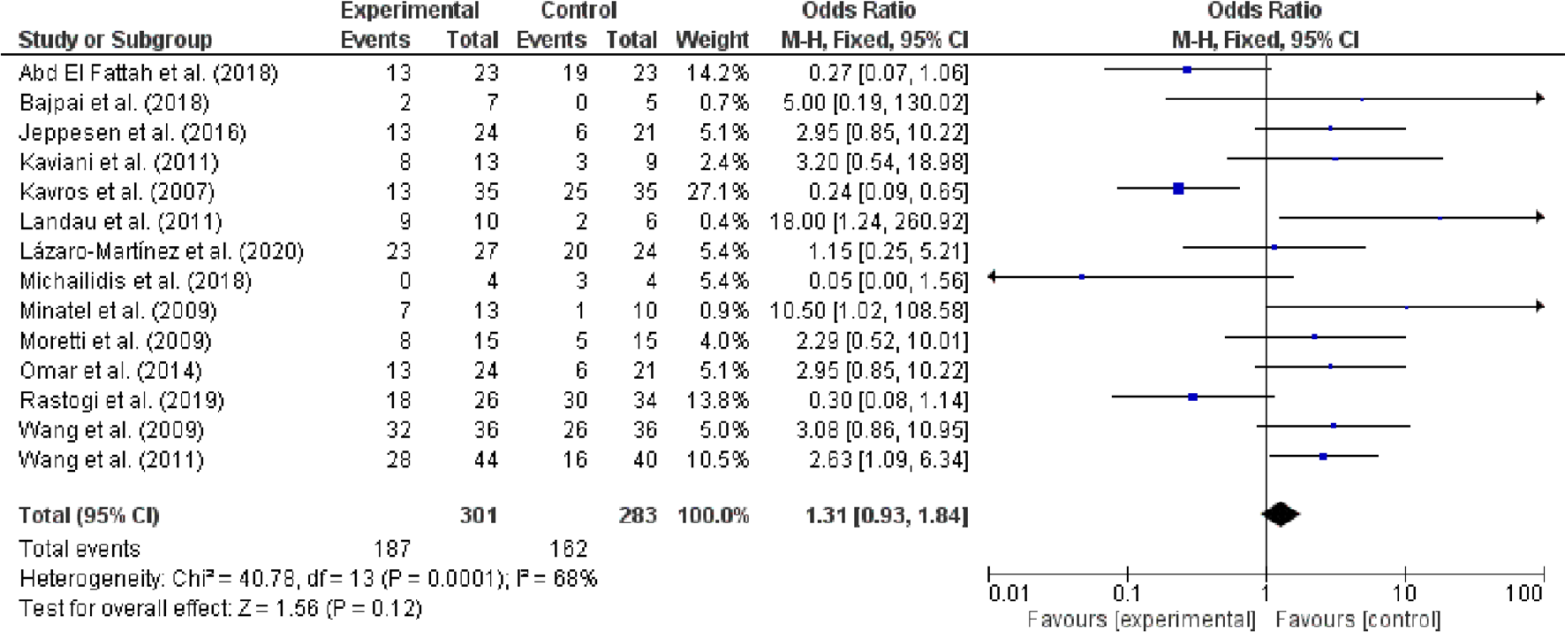
Forest plot showing main event of wound healing among experimental (electromechanical therapies) and control/placebo groups

Similarly, data from fourteen studies compared the number of healed wounds among experimental and control groups. The overall effect was non-significant (P = 0.12) with odds raio (1.31; 95% CI, 0.93, 1.84) for these studies showing better healing among experimental groups compared to control group. There was also a moderate degree of heterogeneity among these studies (I^2^ = 68%, P = 0.00001) (Fig. 3)*.*

## Discussion

In the current study, we looked at novel therapies used to treat DFUs. After a thorough review, extracorporeal shockwave treatment (ESWT) has been shown through experimental research to accelerate the production of angiogenesis-related growth and proliferation factors, shorten the inflammatory phase, and reduce the risk of wound infection [17–22]. Furthermore, by modifying substance P and calcitonin gene-related peptides, ESWT significantly lessens pain in the vicinity of the wound [23]. In 282 patients with chronic wounds who had previously failed conventional treatments, Wolff, Wibmer [18] used ESWT and reported a full cure rate of 74.03% without recrudescence. ESWT was also proven to be effective and well tolerated for treating complex, non-healing, acute, and chronic soft tissue wounds Schaden, Thiele [19]. Thus, ESWT has emerged as a viable first-line treatment for DFU.

In this review, we combined the studies and created a forest plot to compare electromechanical treatments with the placebo/control group. The mean difference for these studies revealed a significant difference between the experimental and control groups, although, the analysis revealed heterogeneity across the groups with a 98% I^2^ value. Overall, there was a significant difference between the experimental and control groups in how quickly DFUs healed. Our findings support those of earlier research by Butterworth, Walsh [24], Dymarek, Halski [25] and Omar, Gwada [26], which prove the effectiveness of ESWT on chronic wounds.

In a randomised clinical trial by Kaviani, Djavid [27], it was shown that low-level laser treatment (685 nm) cured 8 of 13 (66.6%) ulcers in the experimental group compared to 3 of 9 (33.3%) in the control group receiving sham radiation. However, this finding was not statistically significant. In a Level I investigation, Minatel, Frade [28] reported that healing rates for a group of 7 patients with 10 ulcers treated with combined 660- and 890-nm light were considerably greater at 15-day interval than for a group of 7 patients with 13 ulcers treated with placebo radiation. In a recent RCT (Level II evidence), Petrofsky, Lawson [29] found that electrical stimulation and local dry heat resulted in a statistically significant improvement in DFU healing rates and enhanced blood flow to the surrounding area. ESWT resulted in a substantial reduction in wound size and ulcer healing time when compared to normal therapyOmar, Alghadir [30].

Low-level laser therapy (LLLT), one of the adjuvant treatments, has been identified as a viable mechanism of treatment to hasten the healing of ulcers [31]. The findings of several studies looking into the impact of LLLT on DFU are encouraging. Studies have demonstrated a considerable decrease in the size of the ulcer using LLLT with wavelengths of 632 nm (5 J/cm^2^; 20 mW)/904 nm (6 J/cm^2^; 20 mW) and 658 nm (4 J/cm^2^; 30 mW) [30, 76]. In addition to decrease in DFU pains, considerably higher reductions in ulcer size and the percentage of healing compared to controls have been recorded [32–35]. However, its therapeutic advantages rely on a number of factors, making it crucial to identify the best parameterization for the efficient treatment of DFU [36]. Additionally, there is a dearth of reliable data that would support the therapeutic use of LLLT in DFU. The benefits of LLLT on DF have been reported in earlier systematic studies [37, 38], however the current review includes significant updates to its clinical effectiveness and improves parameterization for clinical decision-making.

A prior study [38] suggested the LLLT settings of 660 and 890 nm wavelengths, 50 mW/cm^2^ power density, 2 J/cm^2^ fluence, 30 s of exposure period, and a distance of 1 cm from the wound. The LLLT parameters used in our study were based on the RCTs showing wavelength: 400–904 nm, power density: 30–180 mW/cm^2^, and fluence: 2–10 J/cm^2^. Most of these variables complied with the suggested LLLT settings.

The impacts of LLLT on numerous cellular processes and molecular pathways, such as promoting expression of regulators for cell proliferation, migration, survival, and granulation, were part of the mechanism of LLLT in hastening the healing process of chronic DFU [39]. Additionally, it was discovered that the LLLT group's ulcers had more granulation tissue than the control group [28, 32]. LLLT can increase the expression of essential fibroblast growth factors and induce collagen production in damaged fibroblasts of diabetic mice [40–43]. Transforming growth factor beta [44], interleukin-1 and interleukin-8 [45], platelet-derived growth factor (PDGF)increased macrophage phagocytic activity [46–50]. The synthesis of collagen and extracellular matrix may be increased, the above-mentioned key cytokines and growth factors may be attracted, and the migration, proliferation, and differentiation of various cell types may all be encouraged by LLLT. All these factors may collectively play significant roles in the healing of DFUs. During LLLT therapy, the epithelium and conjunctive tissues displayed unique and quickly expanding cellular renovation, aiding in the process of tissue healing [51]. According to research by Zhou and colleagues, LLLT can increase the expression of heat shock proteins 70 and 1 in injured tissues, which can then increase the synthesis of growth factors like transforming growth factor-beta and aid in wound healing [52].

Regarding the aspect of potential mechanism, it remains unclear. However, the outcomes of the histopathologic analysis show that ESWT can have both a direct and indirect impact. ESWT might encourage collagen production [19], fibroblastic growth, and angiogenesis by increasing cellular ATP production, which then activates purinergic receptors and Erk1/2 signalling [19, 22, 53]. EWST is therefore believed to have the ability to accelerate the healing process. ESWT, on the other hand, may act as a stimulant of microenvironment metabolism and a promoter of dermal cell development, both of which are necessary for ulcer healing. Additionally, ESWT might promote the production of growth factors, such as fibroblast growth factor, transforming growth factor, insulin-like growth factor-1, platelet-derived growth factor, and vascular endothelial growth factor, which are crucial for DFU wound healing [19, 54]. This would then encourage neovascularization of the tissue and enhance blood perfusion.

In terms of safety, electromechanical therapies are acceptable as non-invasive adjuvant treatments. Electromechanical treatments can have adverse effects during treatment, including temporary skin reddening, mild discomfort, and tiny hematomas. Rarely are serious adverse effects and consequences such as bleeding, thrombosis, muscle injury, and wound infections. These show how superior, safety and tolerance of electromechanical treatments are, as well as their potential to be a workable adjuvant therapy for patients with DFU. Haze, Gavish [55] in a study reported that no device-related adverse events were observed in patients with DFU, and percent closure was significantly greater in the active group compared to sham-treated controls.

## Strengths and limitations

The thorough search for evidence, the criteria-based selection of pertinent material, the rigorous assessment of validity, the objective or quantitative summary, and the evidence-based judgments are only a few of this systematic literature review's qualities. The study has several restrictions. The results may only be applicable to people with diabetes and foot ulcers as this meta-analysis only included patients with DFU. Cost-effectiveness was not investigated based on the data available. The included studies' differences in demographic data, baseline ulcer features, and follow-up or treatment periods might possibly have contributed to the heterogeneity observed in the meta- analysis.

## Conclusion

According to the findings of the systematic review and meta-analysis, electromechanical treatments are viable and secure choices for individuals with DFUs. Electromechanical therapy can considerably reduce treatment ineffectiveness, speed up healing, and minimize the time it takes for DFUs to heal.

### Supplementary Information

Below is the link to the electronic supplementary material.

Supplementary file1 (DOCX 14 KB)

Supplementary file2 (PDF 318 KB)

## Data Availability

The datasets used and/or analysed during the current study are available from the corresponding author on reasonable request.
