# Supplementary material for: Electromechanical therapy in diabetic foot ulcers patients: A systematic review and meta-analysis
Source: J Diabetes Metab Disord. 2023 Jun 8;22(2):967–84. doi: 10.1007/s40200-023-01240-2 (PMC10638302; doi:10.1007/s40200-023-01240-2)
Supplement: Supplementary file 1 — Supplementary file1 (DOCX 14 KB) [file 40200_2023_1240_MOESM1_ESM.docx]

## Search strategy

Medical Subject Headings thesaurus (MeSH) terms and keywords from relevant literature were used to build a search strategy that covers all relevant papers.

**Population** (diabetic foot ulcers patients OR diabetes patients)

**Intervention** (Electromechanical therapy OR Laser therapy OR photo therapy OR Ultrasound therapy OR Shockwave therapy)

**Comparator/Control** (Placebo OR Control group)

**Outcome** (Improvement OR Recovery)

Searching whole texts retrieved much irrelevant research so the “abstracts only” approach was used. This type of approach resulted in the acquisition of the most relevant research material. The research was conducted in November, 2022.

PubMed (MeSH) database was used for the search of relevant literature. Screening of titles and abstracts was done in Microsoft Excel. No automated tools were used. The relevancy of the titles, abstracts, and keywords was checked by two independent reviewers. Publications having titles or abstracts that complied with the requirements for this systematic review were chosen for a more thorough examination.
